# Clinical experience on switching trientine tetrahydrochloride to trientine dihydrochloride in Wilson disease patients

**DOI:** 10.1002/jmd2.12451

**Published:** 2024-09-17

**Authors:** Isabelle Mohr, Timo Schmitt, Christophe Weber, Nicolas Schall, Viola Yuriko Leidner, Andrea Langel, Jessica Langel, Aurélia Poujois, Karl Heinz Weiss, Uta Merle

**Affiliations:** ^1^ Internal Medicine IV, Department of Gastroenterology University Hospital Heidelberg Heidelberg Germany; ^2^ Internal Medicine III, Department of Internal Medicine and Cardiology University Hospital Heidelberg Heidelberg Germany; ^3^ Department of Neurology, Rothschild Foundation Hospital National reference center for Wilson disease Paris France; ^4^ Internal Medicine Salem Hospital Heidelberg Heidelberg Germany

**Keywords:** trientine dihydrochloride (TETA 2‐HCl), trientine tetrahydrochloride (TETA 4‐HCl), Wilson disease

## Abstract

This study evaluates the effectiveness and safety of trientine dihydrochloride (TETA 2‐HCl) in patients with Wilson disease (WD) following a switch from trientine tetrahydrochloride (TETA 4‐HCl). A total of 30 WD patients with stable copper metabolism were identified for treatment with TETA 2‐HCl (Cufence™) after prior use of TETA 4‐HCl (Cuprior™). Biochemical markers including urinary copper, non‐ceruloplasmin bound copper (NCC) and liver function were analyzed at baseline and followed up over 12 months. Safety was assessed based on reported adverse events (AEs). Urinary copper levels and NCC remained stable across all follow‐ups, indicating adequate copper metabolism control. Reported AEs during TETA 2‐HCl treatment were mostly gastrointestinal discomfort (*n* = 6). In two patients, progressive elevation of transaminases occurred (despite stable copper metabolism). AEs led to discontinuation of treatment in five cases. Median baseline dose per day was 10.2 mg TETA 4‐HCl/kg bodyweight, whereas median baseline dose after therapeutic switch to TETA 2‐HCl was 12.8 mg/kg bodyweight. Median daily dose at 12 months did not differ significantly from TETA 2‐HCl dose at switching timepoint, with stable biochemical markers and markers of copper metabolism in most (25/30) of the patients. Transitioning from TETA 4‐HCl to TETA 2‐HCl maintained stable copper parameters and liver function in most of analyzed patients. TETA 2‐HCl treatment was generally well tolerated, suggesting that switching medications is safe and effective. In our real‐life cohort, adjustment factor of ~1.25× for the switch of TETA 4‐HCl to TETA 2‐HCl resulted in adequate copper metabolism control.

**Synopsis**

The switch between trientine formulations with different bioavailabilities maintained stable copper parameters and liver function in Wilson disease patients, suggesting that switching medications is safe and effective under controlled circumstances, but conversion dose needs to be adopted individually.

## 1 INTRODUCTION

Wilson disease (WD) is a hereditary disorder of copper metabolism resulting from various mutations in the *ATP7B* gene.^1^, ^2^ Existing guidelines^2^, ^3^, ^4^ outline distinct phases of treatment: initial decoppering and ongoing maintenance therapy. D‐Penicillamine (DPA) was the first oral copper chelator introduced.^5^, ^6^ Subsequently, alternative medications with improved safety profiles emerged, including zinc salts^7^, ^8^ and trientine.^1^ Trientine, initially approved in 1969 for WD patients who could not tolerate DPA,^1^ is now recognized as a viable alternative.^4^, ^9^, ^10^ In 2018, an alternative form of trientine salt (TETA 4‐HCl) was approved. Despite sharing the same active compound, TETA 4‐HCl and TETA 2‐HCl exhibit differences in bioavailability and pharmacokinetics.^11^, ^12^, ^13^ Based on previous pharmacological data it is recommended that when switching patients from TETA 2‐HCl to TETA 4‐HCl a higher dose of TETA 2‐HCl is needed for equal pharmacological effect—due to different bioavailabilities. This dosing recommendation for TETA 4‐HCl is based on data from a randomized, open‐label pharmacokinetic study (TRIUMPH), which involved the administration of a single dose (600 mg trientine base) to healthy volunteers.^14^ The TRIUMPH study revealed a greater bioavailability of TETA 4‐HCl compared to the reference product TETA 2‐HCl.^12^, ^15^ Consequently, an adjustment factor is required, suggesting that approximately 60% of TETA 4‐HCl relative to TETA 2‐HCl should be administered to achieve equivalent trientine base exposure to TETA 2‐HCl: 1 mg of TETA 2‐HCl base is equivalent to 0.6 mg of TETA 4‐HCl base. Subsequent dosage adjustments are recommended based on clinical response and urinary copper excretion. Only limited data on therapy management of patients switching between both trientine formulations is available.^16^ This retrospective study aims to evaluate the switch from TETA 4‐HCl in a real‐world scenario to TETA 2‐HCl with focus on effectiveness, safety and dosing.

## 2 METHODS

### 2.1 Study design

This retrospective, monocentric study examined the clinical and biochemical course of all WD patients in stable therapeutic maintenance phase in 2021 at the University Hospital Heidelberg treated with TETA 4‐HCl (150 mg TETA 4‐HCl base/tablet) to TETA 2‐HCl (200 mg of TETA 2‐HCl base/tablet) with follow‐up (FU) over 12 months after switch. In all cases (*n* = 30) TETA 2‐HCl was chosen as substitute for TETA 4‐HCl due to drug prizing reasons due to the obligation to prescribe the most cost‐effective drug—assuming that both trientine formulations have similar drug actions and are interchangeable. The switch was conducted due to several recourse claims of health insurances experienced in the recent history driven by economic reasons.

Aim of the study was to examine if the therapeutic switch between both trientine formulations is safe and effective. Of note, the study was not performed to detect superiority of one of the trientine formulations. WD patients, diagnosed with WD based on a Leipzig Score of ≥4 (and/or genetic analysis) and demonstrating stable copper metabolism (defined by consistent dosage over 6 months) and stable hepatic and neurologic condition, were included. The baseline for treatment switch was defined as the start of TETA 2‐HCl administration. Due to previous experience at the Department of Gastroenterology and Hepatology of the University Hospital Heidelberg, the cohort was switched with an adjustment factor of 1.25 (Table 5). We aimed to adjust dosage during the FUs depending on urinary copper excretion and liver function tests. Follow‐up assessments were performed at 6 weeks and 3, 6, 9, and 12 months from baseline. The study protocol was approved by the Ethics Committee of the University of Heidelberg (protocol number: S‐565/2011). Written informed consent was obtained from every patient.

### 2.2 Study cohort description

The study cohort involved all WD patients under TETA 4‐HCl in the year 2021. WD patients were instructed to take trientine medication fasted and with a fasting period of 1 h before further food consumption. Compliance with that instruction was checked regularly at each FU. The dose was adjusted during the FUs in accordance with the urinary copper excretion, clinical symptoms and liver function tests. FU visits were performed at 6 weeks and 3, 6, 9, and 12 months from baseline of switching between trientine formulations. Additionally, urinary copper excretion results, laboratory parameters of the liver and transient elastography results were collected.

### 2.3 Statistical analysis

For the statistical data analysis, the pseudonymized Excel database was exported into the statistical software IBM SPSS Statistics (Version 27.0, IBM, Chicago, IL, USA). To depict a primarily descriptive analysis of the study cohort, frequencies, measures of central tendency such as median and mean, as well as measures of dispersion were used. The laboratory parameters during follow‐ups were statistically compared with the paired laboratory parameters at baseline. Mean values of the difference between baseline and follow‐up using a 2‐sample *t*‐test were accompanied by Wilcoxon's rank‐sum test as a non‐parametric alternative, primarily used to assess the potential effect of outliers due to the sample size of the cohort. Median dose was calculated per kilogram bodyweight at baseline and at 12 months to compare conversion factor ad dose adjustments.

## 3 RESULTS

### 3.1 Reasons for discontinuation of TETA 4‐HCl

In all cases, TETA 2‐HCl was chosen as substitute for TETA 4‐HCl due to drug prizing reasons as mentioned above. Chart review confirmed that the switch of TETA 4‐HCl to TETA 2‐HCl was in all cases not related to TETA 4‐HCl associated adverse events or drug failures.

### 3.2 Patient demographics

Mean age of the patients at baseline was 32 years (SD 14.4 years), 46.7% of the patients were male. Mean age at initial diagnosis of WD was 18.5 years (SD 11.9 years). The two pediatric patients were both 17 years old at baseline. In both pediatric patients, transaminases as well as liver synthesis were stable and normal at baseline. Initial diagnosis was made at age of 9 and 11 years. Copper metabolism parameters were stable at time of switch without increased risk of worsening afterwards. Additional individual baseline characteristics are included in the supplemental information (Table 1). In general, previous treatment before switch to TETA 2‐HCl was conducted with TETA 4‐HCl with a median of 28 months (range 8–40 months). None of the included patients was primarily treated with TETA 4‐HCl. Since diagnosis, median treatment duration was 19.3 years (range 2.4–51.1 years).

**TABLE 1:** Patient baseline characteristics.

Parameter	Numbers, medians and percentages (in %)
Number of patients	30 (46.7% male, 53.3% female)
Demographics at baseline	
Age (years)	32 (range 16–61; 2 patients under 18 years)
Weight (kg)	73.8 (range 50–110)
Height (cm)	174 (range 154–197)
Body mass index (kg/m^2^)	23.1 (range 18.1–30.3)
Age at diagnosis (years)	18.5 (range 5–58; 17 patients under 18 years)
Treatment at initial diagnosis	DPA *n* = 26 (87%)
	Trientine 2 HCL *n* = 4 (13%)
Time from diagnosis to recent treatment switch (median, years)	19.3 (range 2.4–51.1)
Previous treatment duration with Trientine 4 HCL (median, months)	28 (range 8–40)
Trientine 4 HCl dosage (median, mg)	735 (range 300–1200)
Clinical presentation of WD	*n* = 22 (73%) primarily hepatic
	*n* = 8 (27%) mixed presentation (hepatic + neurological)
Liver cirrhosis	*n* = 5 (13%, all CHILD Pugh A)
Liver copper (median, μg/g dry liver weight)	1299 (range 253–2727; *n* = 9)
Neurological symptoms	*n* = 8 (27%)
Tremor	*n* = 6 (20%)
Ataxia	*n* = 2 (6%)
Dysphagia	*n* = 2 (6%)
Dysathria	*n* = 1 (3%)
Kayser–Fleischer–Ring	*n* = 8 (27%)

#### 3.2.1 Liver function parameters and liver scores

Liver function parameters remained stable with no statistical differences when comparing each timepoint values with baseline values (Table 2A–E). There were no significant differences with regard to transaminases, cholinesterase, INR, bilirubin, WBC and thrombocytes during the FUs after exposure to TETA 2‐HCl. APRI score and FIB‐4 score act stable during the FU period (Table 3). Stiffness in transient ultrasound elastography did not show any significant differences within 12 months (Table 3). Nevertheless, GGT and CAP appeared to be significantly influenced after therapeutic switch in the 12‐month FU (Table 3). Wilcoxon signed‐rank test only confirmed significant results of the parametric tests for GGT (*p* = 0.028) and CAP value (*p* = 0.024) at 12 months FU (Table 3). Additionally, median BMI was 23.1 (range 18.8–30.3) at baseline and 23.5 (range 18.7–35.6) at 12 months follow‐up. When analyzing the group of patients with previously known liver cirrhosis (*n* = 5) separately, liver function parameters and Child‐Pugh‐score remained stable.

**TABLE 2:** (A–E) Descriptive statistics for key laboratory and score outcomes, baseline, 6 weeks, 3, 6, 9, and 12 months follow‐up.

(A)					
Parameter	Baseline		6 weeks		*p*‐value^b^
	*n*	Median (min‐max)	*n* ^a^	Median (min‐max)	
AST/GOT [U/l]	30	34 (15–58)	15	34 (12–64)	0.955
ALT/GPT [U/l]	30	50 (14–124)	17	47 (20–115)	0.831
GGT [U/l]	30	32 (9–104)	17	32 (13–50)	0.092
Bilirubin [U/l]	30	0.9 (0.4–1.6)	16	0.8 (0.3–1.8)	0.150
INR	30	1.1 (0.9–1.4)	16	1.1 (1.0–1.7)	0.718
Cholinesterase [kU/l]	30	7.8 (4.0–12.6)	16	7.3 (5.1–11.7)	0.155
WBC [/nl]	30	5.0 (2.8–8.1)	19	5.5 (3.3–7.9)	0.147
Thrombocytes [/nl]	30	209 (76–482)	19	216 (88–299)	0.968
24‐h urinary copper [μmol/d]	30	2.6 (0.4–9.25)	16	2.9 (0.85–8.22)	0.959
Serum copper [μmol/l]	30	4.6 (0.9–10.7)	13	4.9 (1.4–11.2)	0.783
FIB4 score	30	1.1 (0.3–3.0)	16	1.0 (0.2–2.4)	0.587
APRI score	30	0.5 (0.2–1.2)	16	0.42 (0.1–0.7)	0.714
Fibroscan					
Stiffness [kPa]	30	8.1 (3.5–21.3)	7	7.9 (3.9–21.8)	0.497
CAP [dB/m]	30	229 (151–324)	7	235 (200–258)	0.176

(B)					
Parameter	Baseline		3 months		*p*‐value^b^
	*n*	Median (min–max)	*n* ^a^	Median (min–max)	
AST/GOT [U/l]	30	34 (15–58)	17	37 (18–61)	0.368
ALT/GPT [U/l]	30	50 (14–124)	17	52 (15–124)	0.756
GGT [U/l]	30	32 (9–104)	17	31 (3–67)	0.476
Bilirubin [U/l]	30	0.9 (0.4–1.6)	17	0.7 (0.1–1.6)	0.064
INR	30	1.1 (0.9–1.4)	17	1.1 (1.0–1.2)	0.852
Cholinesterase [kU/l]	30	7.8 (4.0–12.6)	17	7.6 (4.2–13.3)	0.674
WBC [/nl]	30	5.0 (2.8–8.1)	17	5.6 (3.8–9.1)	0.196
Thrombocytes [/nl]	30	209 (76–482)	17	230 (163–348)	0.469
24 h urinary copper [μmol/d]	30	2.6 (0.4–9.25)	17	2.9 (1.3–10.5)	0.861
Serum copper [μmol/l]	30	4.6 (0.9–10.7)	17	4.9 (1.4–11.2)	0.656
FIB4 score	30	1.1 (0.3–3.0)	17	0.8 (0.4–2.0)	0.943
APRI score	30	0.5 (0.2–1.2)	17	0.4 (0.2–0.8)	0.552
Fibroscan					
Stiffness [kPa]	30	8.1 (3.5–21.3)	17	5.7 (5.2–6.5)	0.593
CAP [dB/m]	30	229 (151–324)	17	177 (132–234)	0.285

(C)					
Parameter	Baseline		6 months		*p*‐value^b^
	*n*	Median (min–max)	*n* ^a^	Median (min–max)	
AST/GOT [U/l]	30	34 (15–58)	23	38 (23–79)	0.330
ALT/GPT [U/l]	30	50 (14–124)	23	55 (14–204)	0.745
GGT [U/l]	30	32 (9–104)	23	36 (14–88)	0.419
Bilirubin [U/l]	30	0.9 (0.4–1.6)	23	0.8 (0.3–1.4)	0.229
INR	30	1.1 (0.9–1.4)	23	1.1 (0.9–1.2)	0.396
Cholinesterase [kU/l]	30	7.8 (4.0–12.6)	23	7.6 (3.9–11.2)	0.136
WBC [/nl]	30	5.0 (2.8–8.1)	23	5.4 (3.3–11.1)	0.605
Thrombocytes [/nl]	30	209 (76–482)	23	220 (87–346)	0.456
24 h urinary copper [μmol/d]	30	2.6 (0.4–9.25)	19	2.3 (0.8–4.9)	0.126
Serum copper [μmol/l]	30	4.6 (0.9–10.7)	20	5.8 (1.5–16.9)	0.085
FIB4 score	30	1.1 (0.3–3.0)	23	1.1 (0.4–2.5)	0.273
APRI score	30	0.5 (0.2–1.2)	23	0,5 (0.2–0.9)	0.436
Fibroscan					
Stiffness [kPa]	30	8.1 (3.5–21.3)	16	7.6 (3.9–25.1)	0.394
CAP [dB/m]	30	229 (151–324)	16	236 (144–330)	0.367

(D)					
Parameter	Baseline		9 months		*p*‐value^b^
	*n*	Median (min–max)	*n* ^a^	Median (min–max)	
AST/GOT [U/l]	30	34 (15–58)	18	36 (26–59)	0.586
ALT/GPT [U/l]	30	50 (14–124)	18	54 (27–157)	0.636
GGT [U/l]	30	32 (9–104)	18	34 (17–67)	**0.023**
Bilirubin [U/l]	30	0.9 (0.4–1.6)	18	0.8 (0.4–1.7)	0.705
INR	30	1.1 (0.9–1.4)	18	1.1 (1.0–1.4)	0.377
Cholinesterase [kU/l]	30	7.8 (4.0–12.6)	18	6.6 (0.9–9.1)	0.272
WBC [/nl]	30	5.0 (2.8–8.1)	18	5.8 (3.6–8.9)	0.006
Thrombocytes [/nl]	30	209 (76–482)	18	220 (116–274)	0.118
24 h urinary copper [μmol/d]	30	2.6 (0.4–9.25)	17	2.6 (0.5–5.3)	0.646
Serum copper [μmol/l]	30	4.6 (0.9–10.7)	18	5.7 (1.1–18.7)	0.209
FIB4 score	30	1.1 (0.3–3.0)	18	1.3 (0.3–3.7)	0.756
APRI score	30	0.5 (0.2–1.2)	18	0.4 (0.2–0.6)	0.885
Fibroscan					
Stiffness [kPa]	30	8.1 (3.5–21.3)	3	7.3 (4.9–11.6)	0.221
CAP [dB/m]	30	229 (151–324)	3	243 (135–380)	0.878

(E)					
Parameter	Baseline		12 months		*p*‐value^b^
	*n*	Median (min–max)	*n* ^a^	Median (min–max)	
AST/GOT [U/l]	30	34 (15–58)	24	41 (24–110)	0.191
ALT/GPT [U/l]	30	50 (14–124)	24	70 (34–302)	0.145
GGT [U/l]	30	32 (9–104)	24	38 (16–89)	**0.028**
Bilirubin [U/l]	30	0.9 (0.4–1.6)	24	0.8 (0.3–1.9)	0.295
INR	30	1.1 (0.9–1.4)	24	1.1 (1.0–1.5)	0.144
Cholinesterase [kU/l]	30	7.8 (4.0–12.6)	24	7.7 (5.6–12.9)	0.199
WBC [/nl]	30	5.0 (2.8–8.1)	24	5.3 (3.5–8.9)	0.278
Thrombocytes [/nl]	30	209 (76–482)	24	210 (89–351)	0.617
24 h urinary copper [μmol/d]	30	2.6 (0.4–9.25)	24	2.8 (0.9–8.8)	0.078
Serum copper [μmol/l]	30	4.6 (0.9–10.7)	24	5.8 (1.7–16.0)	0.163
FIB4 score	30	1.1 (0.3–3.0)	24	1.0 (0.4–2.0)	0.692
APRI score	30	0.5 (0.2–1.2)	24	0.5 (0.2–1.3)	0.291
Fibroscan					
Stiffness [kPa]	30	8.1 (3.5–21.3)	24	7.6 (4.2–21.4)	0.083
CAP [dB/m]	30	229 (151–324)	24	260 (204–357)	**0.024**

Abbreviations: ALT/GPT [U/l], alanine aminotransferase; APRI, aspartate aminotransferase to platelet ratio index: AST [U/L]/(Thrombocyte count [109/L]; AST/GOT [U/l]), aspartate aminotransferase; CAP, controlled attenuation parameter; FIB4, Fibrosis‐4 (FIB‐4) Index for Liver Fibrosis; age [years] × AST [U/L]/Thrombocyte count [10^9^/L] × sqrt (ALT [U/L]); GGT [U/l], gamma‐glutamyltransferase; INR, international normalized ratio; Scale, set of methods underlying the statistical analysis; WBC, white blood cell count.

^a^ Data were not available for all patients at all times.

^b^ *p*‐value was obtained using Wilcoxon rank‐sum test.

**TABLE 3:** Biochemical parameters at initiation, after 6 and 12 months of TETA 2 HCl therapy.

Parameter	Baseline		6 months		12 months		*p*‐value^b^
	*n*	Median (min–max)	*n* ^a^	Median (min–max)	*n* ^a^	Median (min–max)	Baseline vs. 12 months
AST/GOT [U/l]	30	34 (15–58)	23	38 (23–79)	24	41 (24–110)	0.191
ALT/GPT [U/l]	30	50 (14–124)	23	55 (14–204)	24	70 (34–302)	0.145
GGT [U/l]	30	32 (9–104)	23	36 (14–88)	24	38 (16–89)	**0.028**
Bilirubin [U/l]	30	0.9 (0.4–1.6)	23	0.8 (0.3–1.4)	24	0.8 (0.3–1.9)	0.295
INR	30	1.1 (0.9–1.4)	23	1.1 (0.9–1.2)	24	1.1 (1.0–1.5)	0.144
Cholinesterase [kU/l]	30	7.3 (5.1–11.7)	23	7.6 (4.3–11.5)	24	7.7 (5.6–12.9)	0.199
WBC [/nl]	30	5.0 (2.8–8.1)	23	5.4 (3.3–11.1)	24	5.3 (3.5–8.9)	0.278
Thrombocytes [/nl]	30	209 (76–482)	23	220 (87–346)	24	210 (89–351)	0.617
24 h urinary copper [μmol/d]	30	2.6 (0.4–9.25)	19	2.3 (0.8–4.9)	24	2.8 (0.9–8.8)	0.078
Serum copper [μmol/l]	30	4.6 (0.9–10.7)	20	5.8 (1.5–16.9)	24	5.8 (1.7–16.0)	0.163
FIB4 score	30	1.1 (0.3–3.0)	23	1.1 (0.4–2.5)	24	1.0 (0.4–2.0)	0.692
APRI score	30	0.5 (0.2–1.2)	23	0,5 (0.2–0.9)	24	0.5 (0.2–1.3)	0.291
Fibroscan							
Stiffness [kPa]	30	8.1 (3.5–21.3)	16	7.6 (3.9–25.1)	24	7.6 (4.2–21.4)	0.083
CAP [dB/m]	30	229 (151–324)	16	236 (144–330)	24	260 (204–357)	**0.024**
BMI [kg/m^2^]	30	23.1 (18.1–30.3)	16	23.3 (18.8–30.3)	24	23.5 (18.7–35.6)	0.147

Abbreviations: ALT/GPT [U/l], alanine aminotransferase; APRI, aspartate aminotransferase to platelet ratio index: AST [U/L]/thrombocyte count [109/L]; AST/GOT [U/l], aspartate aminotransferase; BMI, body mass index; CAP, controlled attenuation parameter; FIB4, Fibrosis‐4 (FIB‐4) Index for Liver Fibrosis; age [years] × AST [U/L]/thrombocyte count [10^9^/L] × sqrt (ALT [U/L]); GGT [U/l], gamma‐glutamyltransferase; INR, international normalized ratio; Scale, set of methods underlying the statistical analysis; WBC, white blood cell count.

^a^ Data were not available for all patients at all times.

^b^ *p*‐value was obtained using Wilcoxon rank‐sum test.

#### 3.2.2 Assessment of neurological symptoms

All patients were clinically assessed by the same physician at baseline and during the FU with focus on possible neurological worsening. Neurological deterioration during the observation phase was examined through an overall clinical evaluation by the treating physician. None of the patients revealed a neurological worsening throughout the FUs. One patient reported on minimal worsening of the preknown tremor (hand and head), which was no longer present in the 6 weeks FU (Table 5).

#### 3.2.3 Copper metabolism parameters

When comparing copper excretion values of each timepoint with baseline levels at switch timepoint there were no statistically significant differences. Especially the copper related laboratory results (NCC; urinary copper excretion; serum copper) were expected to vary with the factor of time. Neither NCC nor urinary copper or serum copper revealed significant differences within the follow‐ups (Table 4).

**TABLE 4:** Parameters of copper metabolism.

Parameter	Baseline		6 months		12 months		*p*‐value^b^
	*n*	Median (min–max)	*n* ^a^	Median (min–max)	*n* ^a^	Median (min–max)	Baseline vs. 12 months
24 h urinary copper (μmol/d)	30	2.6 (0.4–9.25)	19	2.3 (0.8–4.9)	24	2.8 (0.9–8.8)	0.078
Serum copper (μmol/l)^c^	30	4.6 (0.9–10.7)	20	5.8 (1.5–16.9)	24	5.8 (1.7–16.0)	0.163
NCC	30	1.0 (0.01–3.6)	14	1.0 (0.0–2.0)	15	1.4 (0.01–3.3)	0.678

Abbreviation: NCC, non‐ceruloplasmin bound copper.

^a^ Data were not available for all patients at all times.

^b^ *p*‐value was obtained using Wilcoxon rank‐sum test.

^c^ Urinary copper sampling was performed without discontinuation of medication.

#### 3.2.4 Comparison of TETA 4CHL and TETA 2‐HCl drug dosages

TETA 4‐HCl median baseline dose per day was 735 mg (range 300–1200 mg). This corresponds to 10.2 mg TETA 4‐HCl/kg bodyweight. Median baseline dose after therapeutic switch to TETA 2‐HCl was 907 mg/d (range 600–1200 mg), corresponding to 12.8 mg TETA 2‐HCl/kg bodyweight. Daily dose of TETA 2‐HCl was ≥600 mg in all cases. TETA 2‐HCl median daily dose at 12 months follow‐up was 908 mg (range 600–1200 mg), corresponding to 12.9 mg/kg bodyweight. Comparing median daily dosages and median weight‐adjusted dosages at baseline and at 12‐months follow‐up, median absolute and weight‐adjusted daily dosages did not differ significantly and could not be decreased due to biochemical markers and markers of copper metabolism (NCC, urinary copper excretion). The dose adjustment factor of 1.25 for conversion of TETA 4‐HCl to TETA 2‐HCl resulted in stable copper metabolism parameters and liver function test over the follow‐up period of 12 months, with no signs of progressive copper accumulation after switch.

#### 3.2.5 Safety and treatment discontinuation

There were several adverse events reported as given in Table 5. Nine adverse events were reported within the first 3 months, and five adverse events during the later follow ups >3–12 months in overall six patients. All treatment‐associated adverse events resolved and were mild to moderate. We defined discontinuation of treatment with TETA 2‐HCl due to AEs as of special interest. During the early observation phase of 3 months, none of the subjects discontinued treatment with TETA 2‐HCl. After 3–12 months, five subjects were stopped on medication with TETA 2‐HCl and switched back to TETA 4‐HCl.

**TABLE 5:** Listing of adverse events (AEs).

Adverse events in six subjects	Early (≤3 months), *n* = 9	Late or ongoing (>3–12 months), *n* = 5	Re‐switch to TETA 4 HCl as consequence of AE, *n* = 5
Diarrhea	*n* = 1	*n* = 1^a^	*n* = 1^b^
Nausea (without vomiting)	*n* = 2	*n* = 1	*n* = 1^b^
Acid reflux	*n* = 2	*n* = 1	*n* = 1^b^
Bitter taste	*n* = 1	*n* = 0	
Worsening of tremor	*n* = 1^b^ (patient reported AE, resolved within 6 weeks)	*n* = 0	
Elevation of transaminases			
>1 × ULN	*n* = 0	*n* = 0	*n* = 0
>2 × ULN	*n* = 2^b^	*n* = 0	*n* = 0
>3 × ULN	*n* = 0	*n* = 2^b^	*n* = 2^b^

Abbreviations: AE, adverse event; ULN, upper limit of normal.

^a^ Patient was switched to TETA 4 HCl after 3 months due to ongoing diarrhea.

^b^ Full recovering to baseline status.

The following symptoms occurred newly after switch from TETA 4‐HCl to TETA 2‐HCl and resulted in switching the therapy back to TETA 4‐HCl:

- Persistent diarrhea (5–10 bowel movements/day); *n* = 1
- Persistent nausea; *n* = 1
- Persistent reflux esophagitis symptoms, *n* = 1
- Progressive elevation of transaminases to maximal levels of 3× ULN (*n* = 2). Both subjects (one male/one female) had already slightly elevated transaminases (1–2× ULN) at switch timepoint/baseline. In both cases, elevation of transaminases was resolved to baseline status (elevation to 1–2× ULN) after switching the therapy back to TETA 4‐HCl.

Overall, six patients reported about gastrointestinal symptoms (diarrhea, nausea (without vomiting), acid reflux symptoms, bitter taste). In three cases, these symptoms led to discontinuation of treatment with TETA 2‐HCl (*n* = 2 after 12 months, *n* = 1 after 3 months) and switch back to TETA 4‐HCl. After switch back to TETA 2‐HCl gastrointestinal AEs were reversible afterwards.

In two cases, TETA 2‐HCl was again switched back to TETA 4‐HCl after 12 months due to progressive elevation of transaminases. Copper metabolism measured in 24 h urinary copper was 2.44 μmol/day (patient 1) and 1.23 μmol/day (patient 2) at baseline before switch. Transaminases (GOT [U/l]/GPT [U/l]) were 56/109 in patient 1 and 17/14 in patient 2. At 12 months FU, transaminases (GOT [U/l]/GPT [U/l]) were increased to 110/302 (patient 1) and 67/140 (patient 2). Both were switched back to TETA 4‐HCl after 12 months. In patient 1, elevation of transaminases appeared to be associated to non‐adherence due to reported intermittent nausea after intake of TETA 2‐HCl. 24 h urinary copper was 3.52 μmol/day (NCC 2 μmol/L) in patient 1 at 12 months FU. In patient 2, 24 h urinary copper was 1.44 μmol/day (NCC 0.46 μmol/L) at 12 months FU without known non‐adherence. Liver biopsy revealed normalized liver copper (147 μg/g dry liver specimen). Histopathologically, a drug‐induced or toxic reaction appeared most likely. In both cases, elevation of transaminases was resolved to baseline status after switch of therapy back to TETA 4‐HCl within 6 months. During the 12‐month observation period, one case of early neurological deterioration was a self‐reported AE by one patient during the first 2 weeks after switch. The patient reported about worsening of previously known intention tremor of both hands and head tremor intensity. During the 6 weeks FU, the patient showed a stable neurological condition without worsening of neurological symptoms.

## 4 DISCUSSION

This study represents real‐world data on the switch of TETA 2‐HCl as a substitute for TETA 4‐HCl due to economic reasons in 2021. Previous studies have analyzed the switch of TETA 2 to TETA 4‐HCl.^16^, ^17^ In a retrospective, multicentric study, 68 WD patients treated with TETA 2‐HCl were switched to TETA 4‐HCl. Parameters of copper metabolism as well as liver related outcomes did not reveal a difference to baseline in all FUs. A few AEs during TETA 4‐HCl treatment were reported, most commonly gastrointestinal discomfort. Only three treatments with TETA 4‐HCl were discontinued: One due to a bitter taste in the mouth after 1 week of treatment and two subjects stopped medication due to digestive troubles. Another female subject discontinued after 13 months for progressive elevation of transaminases and was finally diagnosed with a concomitant autoimmune like hepatitis, resolved under immunosuppressive treatment. Another retrospective study was conducted by reviewing data from the national WD registry in France analyzing WD patients who received TETA 2‐HCl or TETA 4‐HCl monotherapy for at least 1 year until 2010.^17^ Ten patients out of 43 had experienced both trientine salts. Direct switch from TETA 2 to TETA 4‐HCl was reported in three patients. All of the patients were clinically stable. No difference in efficacy was detected. Both treatments were well tolerated, except for a case of recurrence of lupus erythematosus‐like syndrome in the TETA 2‐HCl group. The major reason for interruption of TETA 4‐HCl was due to a discontinuation in production of this salt, the reasons for stopping TETA 2‐HCl were mainly due to adherence issues largely attributed to the cold storage requirement.^17^ Especially in the French study, the authors outlined adherence to treatment as a key factor in the successful management of WD. Treating physicians need to be even more vigilant in detecting adherence difficulties in patients after treatment switch.^17^ To our best knowledge our present study examining the switch of TETA 4 to TETA 2‐HCl is the first real‐world analysis of the effectiveness and safety of this switch version. The patient cohort represents a group of WD patients with stable clinical and biochemical situation under TETA 4‐HCl therapy. With a median previous treatment period of 19.3 years (range 2.4–51.1 years) at baseline, the cohort reflects a real‐world cohort of WD patients under maintenance therapy. The research interest was focused specifically on clinical symptoms and occurrence of adverse events, liver function parameters and parameters of copper metabolism. In general, patients revealed effective and safe treatment by TETA 2‐HCl similar to TETA 4‐HCl due to the facts mentioned above. At all analyzed follow‐up timepoints, there were no statistically significant differences in liver function parameters, copper metabolism parameters, APRI and FIB‐4 score when comparing each follow‐up timepoint with baseline data at time of medication switch. The comparison of urinary copper excretion and NCC within the subsets didn't reveal a difference to baseline in all FUs suggesting stable control of copper metabolism under both therapeutic regimes. Nevertheless, Wilcoxon signed‐rank test confirmed significant results of the parametric tests for GGT (*p* = 0.028) and CAP (*p* = 0.024) showing slightly significantly higher values in the 12 months FU after the exposure to TETA 2‐HCl during the observation period. We assume, that the increased GGT and CAP values might be associated to an increasing BMI within the cohort (Table 3) which was observed during the 12 months observation period. The study was performed during the SARS‐CoV‐2 pandemic. It could therefore be possible, that due to the restrictions and less physical activity during that time, patients gained weight which might be reflected in the increased GGT and CAP values. This might only be one possible explanation. Nevertheless, adverse events were reported in six patients. Reported AE's were most commonly gastrointestinal symptoms (diarrhea, nausea [without vomiting], acid reflux symptoms, bitter taste) and elevation of transaminases. Structural neurological examination did not reveal neurological worsening within the observation period. Late neurological deterioration requires careful monitoring in future studies but was not included in this study due to its design limitations. Knowing that long time chelating treatment leads to negative copper balance, it might be difficult to observe hepatic or neurological deterioration within 1 year of study observation.^18^ In 83.3% (*n* = 25) of the patients, treatment with TETA 2‐HCl was effective and safe. 20% (*n* = 6) of the patients developed new adverse events after therapy switch and 17% (*n* = 5) had even to be switched back to TETA 4‐HCl. In our previous study analyzing patients after switch from TETA 2‐HCl to 4‐HCl,^16^ we observed mainly gastrointestinal AEs within the first 3 months in about 41% of the patients. Ongoing AEs (>3 months) were only observed in 6% patients. Therefore, the occurrence of early AEs appears to be higher than of late AEs in both directions of switching trientine formulations. Data verifies that switch of TETA 4‐HCl to TETA 2‐HCl needs to be performed under clinical and laboratory control to detect possible AEs. Under these conditions, it is safe and effective for most of the patients and therefore comparable to the switch of TETA 2 to TETA 4‐HCl.^16^ Non‐ adherence due to possible AEs needs to be monitored carefully, especially when switching between trientine formulation, due to the French registry data.^17^ Nevertheless, the data supports the idea that conversion factor recommended in the TRIUMPH trial (conversion factor: 0.6 for TETA 4‐ to 2‐HCl; 1.6 for TETA 2‐ to 4‐HCl) cannot be one‐to‐one to WD patients. When comparing TETA 4‐HCl (10.2 mg/kg) to TETA 2‐HCl (12.9 mg/kg) dosage in our cohort, the ratio is ~1.25. Our data raises some caution, whether the adjustment factor between both trientine formulations from the TRIUMPH study can be easily transferred to a real‐world cohort of WD patients during maintenance therapy. Potential influencing factors might be different adsorption in WD patients with cirrhosis as well as inadequate adherence and administration twice daily (as in our cohort). Additionally, fasting before intake of medication might be an important confounder of this real‐world study which possibly influences in vivo pharmacokinetic and ‐dynamic parameters. From the pharmacokinetic point of view, the bioavailability of TETA 4‐HCl is superior to TETA 2‐HCl in healthy individuals.^14^ Exact conversion factor has not been analyzed before in a cohort of Wilson disease patients yet. Our findings assume, that adjustment factor between both trientine formulations needs to be adopted individually and might be higher in clinical experience in WD patients than recommended in the TRIUMPH study.^14^ However, the interpretation of the results comes with some limitations due to sample size of the cohort raising some caution concerning the statistical power. This study has certain limitations, including its retrospective monocentric nature and the lack of randomization. Overall, the study delivers relevant data in a real‐world cohort of WD patients on switching trientine formulations. Our findings affirm that efficacy of treatment and copper control under TETA 4‐HCl appears comparable to TETA 2‐HCl. This has already been proved the other way around.^16^, ^17^ Additionally, our data do not reveal a superiority concerning efficacy or safety comparing both trientine formulations. Nevertheless, bioavailability of TETA 4‐HCl could be a pharmacokinetic advantage when it comes to fasting before intake of medication as a possible confounder. Moreover, the necessity of individual dosing is a key factor after therapeutic switch between trientine formulations as well as monitoring of possible AEs. Further randomized studies with longer observation periods are needed to validate our findings.

## 5 CONCLUSION

Our findings affirm that efficacy of treatment and copper control under TETA 4‐HCl appears comparable to TETA 2‐HCl. Switching between trientine formulations needs is safe and effective under clinical and laboratory follow‐ups. The study's limitations preclude establishing superiority between both formulations. Nevertheless, data raises some caution when it comes to conversion factor between both trientine variants. Adjustment factor between both trientine formulations needs to be adopted individually.

## AUTHOR CONTRIBUTIONS

Study concept and design: Isabelle Mohr. Acquisition, analysis, and interpretation of the data: Isabelle Mohr, Timo Schmitt, Christophe Weber, Nicolas Schall, Viola Yuriko Leidner, Andrea Langel and Jessica Langel. Drafting the article: Isabelle Mohr, Aurelia Poujois and Uta Merle. Critical revision for important intellectual content: Isabelle Mohr, Timo Schmitt, Christophe Weber, Nicolas Schall, Viola Yuriko Leidner, Karl Heinz Weiss, Aurelia Poujois and Uta Merle. Statistical analysis: Isabelle Mohr and Timo Schmitt. Study supervision: Isabelle Mohr and Uta Merle. All authors approved the final version of the article and agreed to be accountable for all aspects of the work.

## FUNDING INFORMATION

This study received no funding.

## CONFLICT OF INTEREST STATEMENT

I Mohr advises for Univar and Vivet therapeutics. I Mohr received travel grants from Univar, is Principal Investigator on sponsored studies with Alexion and Univar and received speaker's fees from Orphalan. T. Schmitt, C. Weber, N. Schall, V.Y. Leidner, A. Langel, and J. Langel declare no conflicts of interest. A. Poujois is an advisor for Vivet therapeutics, Alexion, Orphalan and Univar and PI on sponsored studies with Alexion, Univar and Orphalan. K.H. Weiss has received research (institutional) grants from Orphalan; consulting fees from Orphalan, Univar, Pfizer, Alexion, and Vivet Therapeutics; speaker's fees from Falk, AbbVie, Alexion, and Orphalan; travel assistance to meetings from Alexion and Univar; and advisory board payments from Ultragenyx. U. Merle received honoraria for teaching from Falk and Univar. U. Merle received travel grants from Gilead and Falk. All other authors declare no competing interests.

## ETHICS STATEMENT

All procedures followed were in accordance with the ethical standards of the responsible committee on human experimentation (institutional and national) and with the Helsinki Declaration of 1975, as revised in 2000 (5). Informed consent was obtained from all patients for being included in the study. Proof of informed consent is available upon request from the corresponding author (I. Mohr).

## PATIENT CONSENT

Written informed consent for the procedure and management was obtained from all individual participants included in the study.

## Supporting information


**Data S1.** Supporting Information.

## Data Availability

Data are available from the authors with the permission of corresponding author. The data that support the findings of this study are available from the corresponding author (I. Mohr) upon reasonable request.
