# Supplementary material for: Clinical experience on switching trientine tetrahydrochloride to trientine dihydrochloride in Wilson disease patients
Source: JIMD Rep. 2024 Sep 17;65(6):406–16. doi: 10.1002/jmd2.12451 (PMC11540566; doi:10.1002/jmd2.12451)
Supplement: Supplementary file 1 — Data S1. Supporting Information. [file JMD2-65-406-s001.docx]

**Clinical experience on switching Trientine tetrahydrochloride to Trientine dihydrochloride in Wilson disease patients**

**Isabelle Mohr^1^, Timo Schmitt^1^, Christophe Weber^2^, Nicolas Schall^1^, Viola Leidner^1^, Andrea Langel^1^, Jessica Langel^1^, Aurélia Poujois^3^, Karl Heinz Weiss^4^, Uta Merle^1^**

^1^Internal Medicine IV, Department of Gastroenterology, University Hospital Heidelberg, Heidelberg, Germany

^2^Internal Medicine III Department of Internal Medicine and Cardiology, University Hospital Heidelberg, Heidelberg, Germany

^3^Department of Neurology, Rothschild Foundation Hospital, National reference center for Wilson disease, Paris, France

^4^Internal Medicine, Salem Hospital Heidelberg, Heidelberg, Germany

**Corresponding author:**

Dr. med. Isabelle Mohr

Internal Medicine IV

Department of Gastroenterology, University Hospital Heidelberg

Im Neuenheimer Feld 410
69121 Heidelberg
Telefon: +49 6221 56 32818
Telefax: +49 6221 56 5694
eMail: isabelle.mohr@med.uni-heidelberg.de

**Supplemental Information:**

**Baseline characteristics of pediatric patients**

**Table 1:**

| Parameter at baseline | Patient 1 |  | Patient 2 |
| --- | --- | --- | --- |
|  | Female, 9 years old at initial diagnosis, hepatic manifestation at initial diagnosis |  | Male, 11 years at initial diagnosis, hepatic manifestation at initial diagnosis |
| age (years)  weight (kg)  height (cm)  BMI (kg/m2) | 17  62  172  21 |  | 17  55  166  20 |
| TETA 4-HCl dose [mg] | 600 |  | 600 |
| AST/GOT [U/l] | 25 |  | 31 |
| ALT/GPT [U/l] | 20 |  | 25 |
| GGT [U/l] | 30 |  | 40 |
| Bilirubin [U/l] | 0.6 |  | 0.5 |
| INR | 0.8 |  | 0.7 |
| Cholinesterase [kU/l] | 6.5 |  | 5.9 |
| WBC [/nl] | 7.2 |  | 6.4 |
| Thrombocytes [/nl] | 350 |  | 220 |
| 24-h Urinary copper [µmol/d] | 1.1 |  | 1.3 |
| Serum copper [µmol/l] | 5.1 |  | 4.9 |
| FIB4 Score | 0.3 |  | 0.5 |
| APRI Score | 0.1 |  | 0.3 |
| Fibroscan  Stiffness [kPa]  CAP [dB/m] | 7.5  220 |  | 6.5  253 |

**Legend:**

BMI= Body Mass Index; AST/GOT [U/l]= aspartate aminotransferase; ALT/GPT [U/l]= alanine aminotransferase; GGT [U/l]= gamma-glutamyltransferase; INR= international normalized ratio; WBC= white blood cell count; FIB4 = Fibrosis-4 (FIB-4) Index for Liver Fibrosis; age [yrs] * AST [U/L] / (Thrombocyte count [10^9^/L] * sqrt (ALT [U/L]); Scale= set of methods underlying the statistical analysis; APRI= Aspartate aminotransferase to platelet ratio index: AST [U/L] / (Thrombocyte count [109/L]; CAP=Controlled Attenuation Parameter
